# Clinical and laboratory characteristics of COVID-19 among adult patients admitted to the isolation centre at Abubakar Tafawa Balewa Teaching Hospital Bauchi, Northeast Nigeria

**DOI:** 10.11604/pamj.supp.2020.37.1.26162

**Published:** 2020-10-22

**Authors:** Yusuf Bara Jibrin, Okon Kenneth Okwong, Ibrahim Mahmood Maigari, Jacob Amos Dunga, Abubakar Muhammad Ballah, Mustapha Sabo Umar, Alkali Mohammed, Zuwaira Ibrahim Hassan

**Affiliations:** 1 Department of Internal Medicine, Aabubakar Tafawa Balewa University Teaching Hospital, Bauchi, Nigeria,; 2 Department of Medical Microbiology, Federal Medical Centre, Makurdi, Nigeria,; 3 Department of Anaesthesia, University Teaching Hospital, Bauchi, Nigeria,; 4 Department of Community Medicine, Aabubakar Tafawa Balewa University Teaching Hospital, Bauchi, Nigeria

**Keywords:** Nigeria, COVID-19, clinical, laboratory characteristics

## Abstract

**Introduction:**

as the epidemiological trend of COVID-19 infection continue to evolve with increasing prevalence and incidence globally, management of cases in low-resource health care settings require basic detailed clinical and laboratory characteristics. This study retrospectively described the clinical and laboratory characteristics of confirmed COVID-19 cases admitted into the isolation centre of ATBUTH, Bauchi.

**Methods:**

clinical and laboratory data of 84 confirmed COVID-19 cases admitted into the isolation centre of ATBUTH, Bauchi according to NCDC guidelines were used. Diagnosis was based on nasal and nasopharyngeal swab positive result of reverse transcriptase-polymerase chain reaction (RT-PCR) result. Data extracted includes demographic, clinical presentations and laboratory characteristics.

**Results:**

the 84 COVID-19 patients comprised of 72% (59) males and 28% (25) females with mean age of 41.0±10.5 years, majority of the patients were within age-group 21-40 years. Forty-one percent presented with mild to moderate symptoms, 3.6% (3) presented with severe symptoms while 58.3% (49) were asymptomatic with mean body temperature of 36.60C ± Sá. The common clinical manifestations were fever 23.4% (19) and cough 20.7% (17). About 29.3% of the patients had comorbidities, 17.1% (14) were hypertensive while 12.2% of the diabetic. Thirty percent (10) of the patients with DM required intensive care unit (ICU) admission with 10% mortality. Biochemical parameters were within normal range for all the patients. However, haematological parameters showed increased neutrophil (10, 43.5%) and lymphocyte count (19 (59.4%).

**Conclusion:**

the study findings revealed high number of asymptomatic cases, similarity in clinical manifestation and relatively normal laboratory characteristics. More experience with increase in number of patients may provide additional information. Interrupting community transmission will require early detection and contact trace of asymptomatic cases.

## Introduction

The novel coronavirus was reported among patients with unexplained viral pneumonia in Wuhan City, Hubei Province in china in December 2019. Most suspected cases had contact with a sea food market where exotic animals are sold, this is said to have initiated the spread of the global pandemic [1]. The world Health Organization named the disease COVID-19 [2]. Severe acute respiratory syndrome-2 (SARS-CoV-2) belongs to beta coronaviridae family and is associated with severe acute respiratory disease syndrome alongside other members that caused viral outbreak of global public health importance, such as severe acute respiratory syndrome-1 (SARS-CoV) reported in China in 2003 and Middle East respiratory syndrome (MERS) in Saudi Arabia in 2012 and 2013 [3]. Full genome sequencing of early SARS-CoV-2 strain published online showed genetic similarity of 75% to SARS-CoV and 51% to MERS-CoV [4]. The mode of transmission is via respiratory droplets of infected persons and human to human contact, with the transmissibility and infectivity facilitated by the basic reproduction number estimated to be between 2.24 and 3.58 [5]. Documented studies have reported wide range of clinical manifestations with varied severity. Fever and cough are the most common symptoms observed; others include dyspnoea, myalgia, fatigue, nausea and diarrhoea [6-10]. Most studies have shown increased risk of morbidity and mortality amongst elderly patients and those with underlying medical conditions like diabetes mellitus and cardiovascular disease [6-9]. However, emerging trend has also shown increased prevalence of asymptomatic carriers among middle-aged individuals who remain a significant population that increase the likelihood of community transmission [8]. The viral load in asymptomatic patient/carriers is found to be similar to those in symptomatic patients [11], as majority of positive COVID-19 develops mild to moderate cases and some asymptomatic. This trend posed a major challenge to stemming down increasing prevalence and community transmission. Preventive approach remains screening and early detection, isolation, contact tracing and quarantine.

Globally, as at August 2020 there were over 7 million confirmed cases and 600,000 mortality, with varied prevalence influenced by geographical location, ethical/racial and economic class [2]. In Africa, the confirmed cases as at August 2020, were less than 3 million and mortality less than 2,000, a relatively low prevalence compared to other continents. In Nigeria, the first report was an imported case in February 2020, an Italian citizen who arrived Lagos tested positive. In Bauchi State, Nigeria, the first case was confirmed by RT-PCR on 24^th^ March 2020. With border closure and increasing number of confirmed cases indicating local community transmission, early detection, prompt isolation, intervention, contact tracing and quarantining still remain the rational approach to prevention of COVID-19 before and effective vaccine is developed. The laboratory assessment helps in distinguishing moderate, severe and critically ill conditions depending on severity and comorbidities and those requiring ICU admission [12]. Previous studies of severe cases had reported normal/or increased white blood cell count, leucopoenia, lymphopenia, increased haemoglobin level, decreased albumin, increased lactase dehydrogenase, increase CRP, increase D-dimer, aspartate aminotransferase (AST) and alanine aminotransferase (ALT) [6-9,13,14]. In low-resource healthcare setting like ours, COVID-19 infection is additional burden to a weak healthcare system, with lack of basic diagnostic facilities. Therefore, effective management of confirmed cases will require basic clinical and laboratory characteristics of the patients that could serve as template for further management. Hence, we retrospectively described the clinical presentations and laboratory characteristic of 84 COVID-19 cases admitted into our isolation centre at ATBUTH, Bauchi over the 4-month study duration.

## Methods

This retrospective descriptive study examined the 84 confirmed COVID-19 patients data admitted into the isolation centre (ATBUTH) Bauchi between March and June 2020. ATBUTH institutional review board approved the study. Criteria of admission were based on the WHO/NCDC guidelines of positive nasal and nasopharyngeal swab results by RT-PCR result. The clinical classification and management of the patients were based on NCDC National Interim Guidelines for Clinical Management of COVID-19 [15] as follows; asymptomatic infection (patient tested positive for COVID-19, but without manifestations of clinical symptoms or abnormal chest imaging findings), mild cases (patients with non-specific symptoms such as fever, cough, sore throat, nasal congestion, malaise, headache and muscle pain. others include loss of smell, loss of taste, diarrhoea, vomiting and abdominal pain), severe cases (characterized by fever of >38°C or suspected respiratory infection AND one of the following: a) respiratory rate >30 breaths/minute; b) severe respiratory distress; c) SpO_2_<90% on room air). Patients that presented with mild and moderate features were further evaluated and classified according to other associated clinical conditions. Asymptomatic cases were managed in accordance to NCDC guidelines and discharged after returning negative on two occasions. Demographic, clinical and laboratory data extracted from patient medical records include; age, gender, clinical manifestations, comorbidities and laboratory results. Laboratory parameters extracted includes; white blood cell count, haematocrit, neutrophils, lymphocytes, platelets, electrolytes, and liver function tests. Ethical clearance was obtained from the Human Research Ethical Committee (HREC) of ATBUTH. Data was entered into and analysed using SPSS version 20.0. Data was stored in computer with secured pass code. Results were presented as frequencies and percentages.

## Results

A total of 84 COVID-19 patients’ data were analysed, this comprised of 72% (59) males and 28% (25) females. The mean age was 41.5+10.5 years, and majority were within the age-group of 21-40 years, 32 (32.9%). Twenty five (41.7%) patients presented mild to moderate symptoms and 49 (58.3%) asymptomatic cases. Vital signs of the patients showed mean body temperature of 36.6 °C (35-42), pulse rate of 89.7 (18-128), respiratory rate of 22.7 (16-92), systolic rate of 129 mmHg (90-200), diastolic 83 mmHg (60-138) and oxygen saturation rate of 96.5% (58-99). Clinical signs and symptoms, 64 (76.2%) had one signs/or symptoms and 26 (31.0%) had more than one signs and symptoms. Fever 19 (23.4%), and cough 17 (20.7%), were the most common, followed by shortness of breath 9 (11.0%), sore throat and chest pain 4 (4.1%), vomiting 3 (3.7%), diarrhoea and joint pain 2 (2.1%). Comorbidities of hypertension 14 (17.1%) and diabetes mellitus (DM) 10 (12.2%) were the most common. Others includes, HIV 2 (2.4%) and one each for chronic kidney disease (CKD) and asthma 1 (1.2%). Of the 10 DM patients, 3 were within the age group 61-80, required ICU admission, and one died (Table 1). The average hospital stay of the patient was 13 days and clinical outcome showed that 72 (87.8%) were discharged and one mortality. Laboratory characteristics, biochemical parameters showed relatively normal values, hypernatremia 26 (65.0%), hypokalaemia, 11 (28.2%) and increased AST 12 (54.5%) (Table 2). Haematological parameters showed increased neutrophil 10 (43.5%) and lymphocyte count 19 (59.4%) (Table 3).

**Table 1:** demographic and clinical presentation of the patients

Variables	Frequency (%); n=84
**Mean age**	41.5years(4-81)
**Age-group**	
**<**20years	10(12.2)
21-40	32(32.9)
41-60	28(28.0)
61-80	13(15.9)
>81	1(1.2)
**Sex**	
Male	59(72.0)
Female	25(28.0)
**Signs and symptoms**	
Fever	19(23.4)
Cough	17(20.7)
Shortness of breath	9(11.0)
Sore throat	4(4.9)
Chest pain	4(4.9)
Vomiting	3(3.7)
Diarrhoea	2(2.4)
Joint pain	2(2.4)
More than one signs/symptoms	26(31.7)
**Comorbidities**	
Hypertension	14(17.1)
Diabetes mellitus	10(12.2)
HIV	2(2.4)
CKD	1(1.2)
Asthma	1(1.2)
ICU admission	3(3.7)
**Clinical outcomes**	
Discharge	72(87.8)
Death	1(1.2)

**Table 2:** biochemical parameters of the patients

Biochemical tests	Frequency (%)
Sodium(normal 134-145mmol/L)	26(65.0)
Low	26(65.0)
Potassium(normal-3-5.0mmol/L)	26(64.0)
Low	11(28.2)
High	3(7.7)
Bicarbonate(normal-20-30mmol/L)	22(91.6)
Low	1(4.2)
High	1(4.2)
Urea (normal-1.7-9.1g/L)	34(94.4)
High	2(5.6)
Creatinine (normal-72-126umol/L)Low	26(76.6)
Low	4(11.7)
High	4(11.7)
AST-<12	10(45.5)
>12	12(54.5)
ALT<12	23(79.3)
>12	6(20.7)
Total bilirubin<17mmol/l	20(83.3)
>17mmol/l	4(16.7)
Total protein (normal 6.2-8.0g/L)	6(69.4)
Low	2(15.4)
High	2(15.4)
Albumin (normal-2.8-4,9g/L)	5(50.0)
High	5(50.0)

**Table 3:** haematological parameters of COVID-19

Parameters	Frequency (%)
WBC (normal-4-11.0X10^6^)	33(89.0)
Leukopenia	2(5.4)
Leucocytosis	2(5.4)
PCV-anaemia (<33%)	0(0)
Normal	23(0)
Platelets (normal 150-400X10^3^)	23(79.3)
Thrombocytopenia	5(17.2)
Thrombocytocytosis	1(3.5)
Lymphocyte (normal 25-40)	9(28.1)
Lymphocytopenia	4(12.5)
Lymphocytosis	19(59.4)
Neutrophil (normal 40-75)	12(52.2)
Decrease	1(4.3)
Increase	10(43.5)

## Discussion

Globally, the COVID-19 pandemic had revealed vulnerability and unpreparedness of both developing and developing public health care system towards infectious diseases outbreaks, inequality in infection rates and varied pattern in infection rates among nations and communities [2]. In US, high infection rate was observed among population with low economic status and among certain ethic/racial groups [16,17]. In contrast, in sub-Saharan Africa, relatively low prevalence and mortality rates were recorded due to low testing capacity. A high infection rate would worsen an already weak health system with lack of basic diagnostic and management facilities. With limited epidemiological information and available resources, localised information on clinical presentation and laboratory characteristic will be required for effective patient management. Main findings observed were similarities in the demographic and clinical presentations, but difference in the proportion of laboratory values of cases studied. In this study, 35 (39.3%) presented with mild to moderate condition, while 3 (3.6%) were of severe category, 3 (3.6%) severe and 49 (58.3%) asymptomatic. Vital signs recorded were normal with mean body temperature of 36.6°C. Globally, the body temperature check had been used as screening indicator and monitoring of suspected individuals at point of entry to school, hospital and airports. Consistent with other studies, the relatively normal body temperature recorded among most asymptomatic individuals might not be a good indicator as many positive asymptomatic individuals will be missed unless RT-PCR testing is carried out for suspected cases and during contact tracing as observed in our study. Fever, cough and dyspnoea were the commonest symptom at presentation, others included loss of taste, smell, fatigue and diarrhoea. This is similar to reports in similar publications (in China, Europe and US) [18,19]. Previous studies had reported wide range of clinical manifestation with fever, cough and dyspnoea as the most common, others include loss of taste, smell, fatigue and diarrhoea dependent on severity of infection [7-9].

In this study, 62 (73.8%) presented one signs/or symptoms, while 26 (31.0) patients had more than one signs and symptoms. Fever 19 (23.4%) and cough 17 (20.7%) accounted for most clinical manifestations, consistent with other studies, which also includes, shortness of breath, sore throat, chest pain vomiting and diarrhoea depending on severity of infection [6,7]. In Malaria endemic region like Nigeria, the use of fever as leading clinical indicator of COVID-19 infection may be a misleading indicator in some cases as fever is a common clinical presentation at hospital and in communities, which is often treated with antimalarial drugs. Moreover, diagnosis of COVID-19 is limited to few designated molecular laboratories in the country. Adopting routine haematological indices for triaging patients with fever for early detection becomes necessary. A study conducted in China among COVID-19 patients presented at fever clinic [20], suggested the use of eosinophilia and CRP, which showed relatively high sensitivity and specificity for detection and isolation.

Earlier studies on COVID-19 had observed high infection rate among the elderly, but emerging trend showed increased prevalence among middle-age population with asymptomatic presentation [21,22]. In this study, the mean age of the 84 patients was 41.0 years, lower than range of 45-65 years reported in most studies [6,9,14], but comparable with some studies, 38 years in Lagos [10] 36 years in Oman [23] and, 41 years in China [24]. Most studies had reported male gender predominance [6,12], which collaborate with study of 72% males and 28% females. Higher expression of angiotensin-converting enzyme-2, sex hormones, innate and adaptive immunity had been suggested as factors responsible for low susceptibility of females to the infection [25]. However, outdoor socio-cultural and religious activities allow for crowding and clustering that could facilitate exposure to the virus and community transmission. Comorbidities, particularly individuals with chronic illness are associated with COVID-19 infection that influenced the severity and outcome [9,21]. Hypertension and diabetes mellitus were the most common comorbidities associated with severe COVID-19 infection, similar to comorbidities of our patients, hypertension 14 (17.1%) and DM 10 (12.2%) though lower proportion due to the composition of our patients [9,26]. However, the three severe cases were diabetic patients within the age-group 61-80 years that required ICU admission and one died.

Laboratory parameters helps in distinguishing mild and moderate from severe, and those required ICU admission, as the values varies between severe and non-severe cases. Normal/or elevated white blood cell (WBC) counts, lymphopenia, thrombocytopenia and leucopoenia, have been reported among severe cases requiring ICU care with clinical outcome [6,13,14,24]. Elevated CRP, ALT, neutrophil, urea, decrease albumin, lymphopenia, and IL-6 were also observed in worsening cases resulting in mortality [12,27], indicating progression into critically level with resulting liver and renal function test derangement [12,14,24]. Laboratory characteristics of our patients showed relatively normal biochemical values with slight increase in AST level. However, haematological parameters showed increased neutrophil counts and lymphocytosis. This indicates underlying infection collaborated by other studies that revealed COVID-19 patients can present confected with other respiratory pathogens -viruses, bacteria and Fungi [9,28]. The study findings had provided a baseline information on clinical presentation and laboratory characteristics of COVID-19 cases necessary for better assessment and management in low resource health care setting. Despite the significance of the study findings there are limitations which include limited clinical and laboratory information, composition of patients and non-uniformity on laboratory tests carried out on the patients. Radiological appearance could have also suggested severity of disease at presentation.

## Conclusion

The study findings revealed similarity with other studies in the epidemiology as relates to the demographic pattern but differs in laboratory characteristics due to the composition of COVID-19 cases. The high proportion of asymptomatic cases posed a major challenge to the control and stemming down infection rate in the community. Further study of larger sample size with regional outlook, radiological investigations and effect of early intervention and outcome is suggested.

### What is known about this topic

- Epidemiological, clinical and laboratory characteristics of COVID-19 among adults is lacking in the study area since the index cases was reported;
- Relatively few documented studies are available for evaluation and assessment of epidemiological situation.

### What this study adds

- The study revealed demographic, clinical presentation and laboratory characteristics of COVID-19 in the isolation centre;
- The composition of patients was made up of relatively few severe cases and high proportion of asymptomatic cases;
- Laboratory parameters revealed relatively normal values except two haematological indices.
